# Inside the Black Box: What Makes SELEX Better?

**DOI:** 10.3390/molecules24193598

**Published:** 2019-10-07

**Authors:** Natalia Komarova, Alexander Kuznetsov

**Affiliations:** Scientific-Manufacturing Complex Technological Centre, 1–7 Shokin Square, Zelenograd, Moscow 124498, Russia; kae@tcen.ru

**Keywords:** aptamer, SELEX, nucleic acid library, PCR amplification, ssDNA regeneration, sequencing, next generation sequencing

## Abstract

Aptamers are small oligonucleotides that are capable of binding specifically to a target, with impressive potential for analysis, diagnostics, and therapeutics applications. Aptamers are isolated from large nucleic acid combinatorial libraries using an iterative selection process called SELEX (Systematic Evolution of Ligands by EXponential enrichment). Since being implemented 30 years ago, the SELEX protocol has undergone many modifications and improvements, but it remains a laborious, time-consuming, and costly method, and the results are not always successful. Each step in the aptamer selection protocol can influence its results. This review discusses key technical points of the SELEX procedure and their influence on the outcome of aptamer selection.

## 1. Introduction

Aptamers are small synthetic nucleic oligonucleotides that serve as ligands to target molecules. With their small size, low synthesis cost, thermal stability, ease of labeling, and ability to regenerate, aptamers are superior to antibodies and have gained high popularity as a tool for analysis and diagnostics and as therapeutic agents [1]. The range of aptamer targets is strikingly large, starting from small molecules through proteins and viruses to whole cells [2,3].

Aptamers against a particular target can be obtained by the iterative selection of target-binding species from a large library of random oligonucleotide sequences. This procedure, called SELEX (Systematic Evolution of Ligands by EXponential enrichment), was first proposed in 1990 simultaneously by Ellington and Szostak [4] and Tuerk and Gold [5]. Throughout the last three decades, many variations of this protocol have been applied thousands of times to obtain an increasing number of aptamers. The aim of most of these studies has been the practical implementation of an aptamer, and they have used SELEX as a “black box” instrument, paying more attention to the detailed characterization of novel aptamers and the development of their applications. Investigating the SELEX process itself is not as popular a task, but the information derived from such studies can help to increase the efficiency of aptamer selection. Two main features can be highlighted as the parameters that characterize the efficiency of the SELEX procedure: The properties of the selected oligonucleotides, such as affinity and specificity, and the labor efforts for aptamer selection, indicated foremost by the number of selection cycles required for successful aptamer isolation. Besides this, overall selection success is a kind of fortune, as far as sometimes the nucleic acid library is lost during the aptamer selection due to the absence of target-binding species in the initial library or some technical difficulties of the experiment. This review summarizes comparative and elucidating studies on the SELEX process and discusses the impact of experimental issues on the selection outcome.

## 2. The Conventional SELEX Protocol

**Figure 1:**
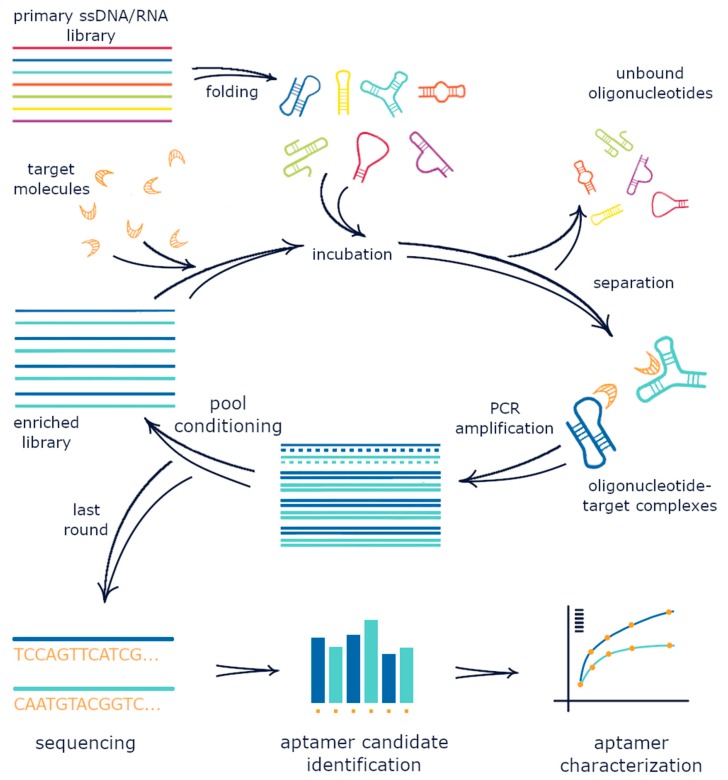
Illustration of the SELEX procedure.

A SELEX experiment starts with the preparation of the nucleic acid library. First, a large random RNA or DNA pool is incubated with the target molecule. In most selections, target molecules are immobilized onto a carrier to enable further separation of oligonucleotides, which fold into a complex with the target, from non-binding species. The target-bound nucleic acid pool is then amplified by PCR. ssDNA or RNA is further regenerated from the PCR products to produce a new oligonucleotide pool with an increased fraction of nucleotides that are able to bind to the target. This pool is again subjected to a new selection cycle. Iterations of binding, separation, amplification, and pool conditioning are repeated until the resulting nucleic acid pool is enriched enough with target-binding sequences (see Figure 1 for illustration). Typically, 5–15 selection rounds are required. The enriched pool is sequenced, and aptamer candidates are identified from the sequencing results using bioinformatics analysis. The selected sequences are chemically synthesized and characterized by their binding ability and specificity to a target. Experimental details can influence the selection speed and success of the process, as well as the affinity and specificity of the obtained aptamers [6,7,8,9]. 

## 3. Library Design

The starting library of oligonucleotide sequences is essential for the success of aptamer selection. The classic nucleic acid library consists of a random core of 20–60-nt-long species flanked by fixed regions that are used for library reamplification. Having the maximum possible diversity in the initial nucleic acid library is crucial for selection success because it directly influences the probability of the presence of target-binding sequences in the initial oligonucleotide pool.

The quality of the nucleic acid library depends on many parameters, starting with chemical synthesis. During phosphoramidite-based synthesis, nucleotides are unequally incorporated into an oligonucleotide chain, with a preference for G and T. For equal nucleotide distribution, the molar ratio of A:C:G:T phosphoramidites should be optimized—for example, a 1.5:1.5:1.0:1.2, 1.30:1.25:1.45:1.00, or 1.50:1.25:1.15:1.00 A:C:G:T molar ratio can be applied [10]. The same libraries obtained from different manufacturers can differ significantly in sequence heterogeneity and, consequently, in structure complexity, which has been proved by the analysis of high-throughput sequencing results [11].

The quality of the nucleic acid library can be assessed prior and during the SELEX experiment with several methods. The correct length and purity of the library can be validated with electrophoretic separation-based techniques (using bulk gel separations or capillary electrophoresis-based equipment like the 2100 Bioanalyzer offered by Agilent [12]). The library diversity can be evaluated with melting and remelting curve analysis [13,14,15] or next generation sequencing [11]. qPCR and fluorescent dyes-based measurements serve for precise quantification of libraries. Computer modeling algorithms like mfold [16] are employed for prediction of possible secondary structures of the library species.

The key aspects of nucleic acid library design were thoroughly reviewed by Vorobyeva et al. [17]. All of these factors can alter the selection process to varying extents.

### 3.1. The Length of the Random Region

The size of the random core of the oligonucleotide library most notably defines the library diversity, which in turn contributes to the success of the overall aptamer selection. The larger the library diversity, the higher the chance that oligonucleotides displaying target affinity are present in the library. Theoretically, a longer random region enables a higher number of possible sequences that can be obtained. In practice, it is generally believed that a library comprising 10^15^ molecular species is enough for successful aptamer isolation and, at the same time, is relatively simple to obtain during chemical synthesis [10,18,19]. This size corresponds to the length of the randomized region of 25 nt. Libraries with a larger randomized region are not fully represented, but employing larger libraries is still worthwhile because such libraries display a larger diversity of three-dimensional (3D) structures of both DNA and RNA oligonucleotides [19,20].

The influence of the randomized region length on in vitro molecular evolution was investigated in a series of research works on the isolation of isoleucine-binding RNA aptamers [21,22] and ribozymes with thioester-synthesizing [23,24] and self-cleaving activity [20].

The length of the randomized region can affect the selection with respect to both the possibility of successful aptamer isolation and the selection speed. An isoleucine-binding RNA motif of 16 nt in length was isolated within 5 selection rounds using a starting library with a random region of 26 nt and within 7 rounds using a starting library with a random region of 22 nt, and it was not isolated at all within 11 selection cycles using a short 16 nt random region library [21]. The reason for these results is that, in a more extended library, the short binding motif can appear more frequently than in a shorter library. Extending this experiment, Legiewicz et al. [22] compared libraries with a random core of 16, 22, 26, 50, 70, and 90 nt for the selection of an isoleucine-binding motif, and it was isolated with the maximum speed from libraries with 50 and 70 nt randomized regions. The selection of a self-cleaving hammerhead ribozyme using a relatively long RNA library enabled the isolation of a few ribozyme types [20], while a shorter library led to the isolation of only the simplest of possible motifs [25]. Even longer self-cleaving ribozymes exist that can be isolated only by using longer libraries [20] because the possibility of finding a long and complex motif in a short library is rather low. Besides this, random regions that are too short tend to interfere with the fixed regions to a greater extent than larger ones [26].

To our knowledge, no similar investigations of library length impact to selection process with implementation of SELEX experiments have been performed using DNA libraries.

The library length impacts not only library diversity and motif representation, but also the experimental procedures coupled to SELEX. In particular, the efficiency of RNA regeneration depends strongly on its length. During the isolation of an isoleucine-binding aptamer from a mixed RNA library with a random region of 30, 60, 100, and 140 nt in length, the shorter oligonucleotides were more able to survive [23]. Longer libraries with 140 and 100 nt cores were lost, and at the end of the selection process, oligonucleotides with a 30 nt random region were much more abundant than those with 60 nt. Shorter DNA fragments are transcribed to RNA with substantially higher efficiency and fewer errors [23,24]. In addition, long RNA ribozymes are not much more efficient than short ones, and the likelihood that they misfold into inactive conformations increases with their length [24]. As for DNA, PCR amplification of longer heterogeneous DNA templates is encumbered with byproduct formation to more extent than amplification of shorter libraries; namely, byproduct formation starts with less amplification cycle numbers for longer matrix, and higher dsDNA product yield is achieved for shorter ones [27]. Shorter aptamers are preferable for most practical applications; thus, numerous post-SELEX experiments have aimed to minimize the aptamer structure. Finally, the oligonucleotide yield during chemical synthesis decreases as the oligonucleotide length increases, and the synthesis of longer oligonucleotides is more labor-consuming and cost-intensive [10].

Therefore, an optimum length of the randomized region can be suggested to exist, given the experimental results described above. If the random core is too short, the binding motifs can appear too rarely or even be nonexistent in the starting oligonucleotide pool. For libraries that are too large, library synthesis and conditioning encumber the selection process. 

### 3.2. Primer-Binding Site Sequences

With a randomized region length of 20–60 nt, flanking constant regions of 18–21 nt each comprise 40–70% of the total library structure. Consistently, primer-binding sites can be assumed to influence the aptamer structures and selection process. These constant regions can form self-dimers and primer dimers that predefine library structures and thus reduce the diversity of the starting library, or they can interfere with the central core to block binding motifs with duplexes and shift the evolution of the oligonucleotide pool [18].

Bioinformatic evaluation of the contribution of constant regions to aptamer structures by analyzing over 2000 aptamers of 141 targets showed that primer-binding sites are minimally involved in the overall structures of the selected aptamers [26]. Indeed, several aptamer truncation experiments have confirmed that the excision of primer-binding sites and the further shortening of the aptamer sequence often allow for the attainment of a simpler structure with the same or even better affinity to the target [28,29,30,31]. However, several comparative experiments have led to the opposite conclusion. In [32], three selection strategies for genomic SELEX (gSELEX) were compared to assess the contribution of primer-binding sites: Conventional gSELEX with fixed flanking regions, selection employing complementary sequences to block primer-binding sites, and selection with the complete switching of primer sequences during the SELEX process. Fixed regions were shown to affect the selection outcome by producing artifact oligonucleotides that lacked binding ability [32]. Another comparison of gSELEX strategies—conventional, primer-blocking, and primer-free—indicated that the selected sequences differ strongly depending on the selection type, and oligonucleotides selected through the use of fixed regions can lose affinity to the target if the flanking constant sequences are truncated [33]. During the isolation of the above-discussed isoleucine-binding motif, a set of libraries with two different primer-binding sites was used. In both cases, the same motif was successfully isolated, but the constant regions affected the structural diversity of the selected RNA pools. If primer-binding sites interfere with the binding motif, the isolation of the latter is obstructed [22]. In another selection experiment, alternate primer sequences resulted in the isolation of a different motif that could bind to isoleucine [34]. A constant 5’-end region was shown to define the sequence of an RNA aptamer of arginine [35]. In their stem–loop structures, all duplexed nucleotides were complementary to the primer-binding site, stabilizing the whole aptamer structure, while nucleotides located in loops were found to interact with the target. Although nucleotides that are responsible for target binding have been evolutionarily defined by the target, the influence of the constant regions on the selected sequences is apparent. Because of the influence of fixed regions, conventional SELEX is considered to be irrelevant for modeling the evolutionary process with in vitro selections [36]. The negative influence of primer sequences is also undesirable in genomic SELEX in vitro experiments that aim to identify the DNA–protein interactions that take place in vivo [32,37].

Besides these challenges, primers can contribute to the formation of byproducts during the PCR amplification step [27], resulting in the retardation or failure of selection, and this problem can be overcome with accurate primer design [38].

Experimental evaluation of primer-binding sites’ contribution to aptamer selection was performed by Yang et al. [39]. Three N_30_ DNA libraries, referred as library A, B, and C by the authors, were used in parallel to isolate aptamers against a protein target (alphamethylacyl-CoA racemase, AMACR, which is a cancer marker). Primer-binding sites were designed to form different 3D structures. Flanking regions in libraries A and B formed a stem–loop structure on both sides, while the primer regions of library C were partially complementary to each other and thus could help the library oligonucleotides to fold into hairpin structures. For these libraries, different structural composition and diversity were proved experimentally by CD spectra and melting curve analysis. Four rounds of selection were performed using these libraries. Primer-binding sites affected evolution rates during SELEX, and due to that, different selection cycle numbers were required to gain maximum binding of the pool oligonucleotides to the target. Representatives of each pool were Sanger sequenced, and the resulting sequences were all different. For each library, two aptamer candidates were characterized. Aptamers derived from each library differed in affinity and specificity to the target. Library A resulted in aptamers with better K_D_ value but lacking specificity. Aptamers from libraries B and C exhibited slightly less well affinity but demonstrated good specificity to AMACR. Aptamers originating from libraries A and B were shown to be pre-folded, while aptamers from library C showed structure-switching properties upon binding to the target. A truncation experiment for the selected aptamers was performed to evaluate the effect of constant regions. Trimming of primer-binding sites caused strong decrease of affinity for aptamers derived from libraries A and B, and had practically no effect on aptamers from library C. The authors supposed that this was due to initial design of primer-binding sites. As fixed regions in library C were partially complementary to each other, this prevented their hybridization to the random region and thus minimized participation of primer-binding sites in aptamer function [39]. This work clearly demonstrates the impact of primer-binding sites to selection results and the importance of appropriate design of priming regions.

Special selection approaches have been developed to prevent or minimize the possible influence of primer-binding sites on the selection process. Primer-eliminating and primer-minimizing methods have been developed for genomic [33], RNA [40,41,42,43], and DNA [32,44,45] oligonucleotide libraries. Primer switching during the selection process can also be used to reduce the influence of fixed regions [46]. Most of these protocols have been observed and classified in specific reviews [17,37]. All of these procedures are much more time- and labor-consuming because of the introduction of additional operations, but they have been shown to result in successful aptamer selection. Another approach is based on specially constraining the structures of the fixed regions so that they fold into self-blocking duplexes [43,47], although this can sometimes predefine the library structure and thus lower its diversity. Finally, the simple blocking of primer-binding sites with complementary oligonucleotide strains can be applied to minimize the influence of primer-binding sites on library folding [7,32,33,48,49]. This technique has been experimentally proved to significantly decrease the probability of the loss of target-binding sequences in the first selection cycle and to reduce the number of selection rounds needed for the isolation of aptamers with high affinity [48], although it still biases the selection more than primerless and primer-switching strategies [32,33]. 

In summary, interference between randomized and constant regions and the degree of its negative influence on the results of aptamer isolation seem to be case-specific but definitely worth special attention because the effects cannot be predicted before the selection. Libraries with a shorter randomized region tend to interfere with constant regions to a greater extent [26].

### 3.3. Structural Features of the Library

Uniform nucleotide distribution in the random core is usually believed to ensure the maximum structural diversity of the library. However, computation analysis of random RNA pools has revealed that complex branched structures are relatively rare in such pools [50], and specially designed libraries are required to achieve higher oligonucleotide content with more complex structures. Within a relatively short random RNA library core, higher GC content was shown to provide a larger number of oligonucleotides with more complex structures, although this contention is not applicable to longer libraries [51]. G-rich libraries result in a higher number of G-quadruple structures [52]. It must be noted that the key to selection success using non-uniform libraries lies more in structural diversity than structural complexity [53]. Specially developed computational methods can assist in the design of more structurally diverse libraries [53,54,55,56].

Patterned libraries with special nucleotide distributions [57] and partially structured libraries with predefined stem–loops [58] and three-way junctions [59,60] have been successfully implemented in SELEX and have proved to be efficient for aptamer isolation.

Patterned libraries and libraries with predefined structures have been experimentally proved to be superior to conventional libraries with uniform nucleotide distributions. Ruff et al. [57] performed competitive selections with protein targets using a mix of a uniform library and two types of patterned DNA libraries with purine and pyrimidine interlacing motifs. For two targets streptavidin and IgE, one of the patterned libraries provided greater enrichment after 9 or 10 rounds of selection. This was attributed to the higher content of potent sequences in this library compared with the others. Aptamers obtained from this library also demonstrated a higher affinity to the target [57].

In another competitive selection experiment, the introduction of a predefined stem–loop to the center of a random RNA library core enabled the isolation of GTP-binding aptamers with higher affinity to the target compared with a non-structured library [51]. Later, different GTP-binding aptamers were compared, and among them, aptamers with more complex structures possessed a higher affinity to the target [55]. Moreover, intramolecular RNA interactions were found to contribute to this increase in affinity to a greater extent than target–RNA interactions [58].

Taken together, these results suggest that the use of specially designed non-uniform libraries enable the isolation of aptamers with higher affinity.

### 3.4. Chemical Modifications of Nucleic Acids

Chemically-modified nucleic acids have gained popularity during the last decade [17]. Two objectives are usually pursued with these modifications: (1) The improvement of nuclease stability of aptamers and (2) the expansion of chemical functionality of nucleic acids. This field of aptamer development is rather expansive and has been extensively discussed in specialized reviews [61,62,63,64,65]. 

Modifications of sugar–phosphate backbone are used to protect aptamers from nuclease degradation that is especially relevant for medical application as it allows increasing of aptamer half-life in the biological media [61]. Substitution of 2’-OH group in RNA ribose with 2’-F, 2’-NH_2_, 2’-O-CH_3_, 4′-S- ribose modification in RNA, replacement of sugar moiety of nucleic acid with locked ribose (locked nucleic acid, LNA), acyclic carbohydrates (unlocked nucleic acid, UNA), threose (threose nucleic acids, TNA), fluoroarabinose (fluoroarabino nucleic acids, FANA), changing phosphate to methylphosphonate, phosphothioate and even triazole, 3’- and 5’- end labeling of aptamers, chiral transition of nucleic acids (spiegelmers)—all these chemical modifications are successfully applied to obtain aptamers with improved thermal stability and nuclease resistance using both initial selection from modified libraries or post-SELEX modifications [61,62]. Mutant enzymes are required to perform selections with backbone-modified nucleic acids, as commonly used natural polymerases are incapable to proceed with xeno nucleic acids [63], with a small exception for 2’-F RNA, which can be handled with standard T7 RNA polymerase [17]. 

Modification of heterocyclic bases with different (preferably hydrophobic) moieties serves to expand the alphabet of nucleic acids, which, in turn, results in uprising of new protein-like binding abilities for the aptamers. Base-modified aptamers often exhibit higher affinity to the targets compared to aptamers selected from non-modified nucleic acids [66,67]. SOMAmers (Slow Off-Rate Modified aptamers) are a very popular example of such base-modified nucleic acid-based aptamers with superior affinity to the targets [68,69]. Besides higher affinities, base-modified aptamers exhibit a wider target range and higher nuclease stability [69]. The base-modified nucleic acid libraries for aptamer selections are generated using two different strategies. The first way is enzymatic incorporation of pre-modified nucleotide triphosphates during library synthesis. Implementation of unnatural nucleotides to SELEX in this case also demands usage of specially engineered enzymes capable of incorporating them into the nucleic acid chain in primer extension reaction instead of PCR [63,70]. One more method is click-SELEX, which is used to generate base-modified libraries after the synthesis of the nucleic acid chain. For this purpose, thymidines in the nucleic acid chain are substituted with C5-ethynyl-2’-deoxyuridine, which is further modified using click reaction with copper(I)-catalyzed alkyne–azide cycloaddition [61]. This method is compatible with commonly used enzymes.

Aptamers derived from chemically-modified nucleic acids definitely offer benefits over natural DNA/RNA aptamer in terms of affinity, selectivity, and stability, but the SELEX protocol for their isolation is much more complicated [17,63].

## 4. Selection Conditions

### 4.1. Target (Amount and Concentration)

The target concentration in the binding step is an essential parameter of selection. A number of SELEX mathematical models have been developed [71,72,73,74,75,76,77,78,79]. All of these modeling studies have paid significant attention to target concentration and have often provided the equations required for its calculation.

Almost all of the modeling research has postulated the existence of the optimal target concentration, which depends on the concentration of the oligonucleotide pool, its bulk dissociation constant, the partition efficiency of the selection protocol [71,72,73,74,75,76], and even the number of PCR cycles in the pool amplification step [74]. With a lower target concentration, the probability of isolating ligands with high affinity increases. The higher the target concentration, the greater the possibility of isolating ligands with lower affinity. However, if low-affinity ligands predominate over high-affinity ones in the oligonucleotide pool, then those with low affinity tend to be selected better with a low target concentration [72,73,74]. This means that a very low concentration of the target is better for selection only in theory; in practice, low-frequency high-affinity oligonucleotides are isolated in small amounts and can be easily lost during pool amplification and conditioning [74]. Modeling experiments have shown that with a reduced target concentration, the number of selection cycles required for the isolation of high-affinity oligonucleotides first decreases, but then starts to increase [72]. In [76], the optimal target concentration was reported to be equal to the bulk *K_D_* of the oligonucleotide pool. For the best results, accurately decreasing the target concentration during selection is advised, and the bulk *K_D_* measurements of the selected pool are needed to assess the optimal concentration [72].

Another model specifically illustrates the dependence of the optimal target concentration on partition efficiency [75]. In an ideal selection, only affinity ligands are isolated in each selection cycle, and a lower target concentration enables better library enrichment, leading to the minimization of the number of selection cycles. In practice, a reduction in target concentration becomes senseless from the very moment it becomes equivalent to the *K_D_* of high-affinity ligands in the oligonucleotide pool, because a further decrease in concentration results in only a slight improvement in the enrichment. With increasing background binding, the library enrichment is impaired, and the benefit gained from optimizing the target concentration to provide maximum enrichment emerges [75]. On the basis of this model, two SELEX experiments with high (microfluidic platform selection) and poor (nitrocellulose membrane selection) partition efficiencies were simulated, and the selection with higher background binding was shown to be much more sensitive to target concentration [75]. Interestingly, the results of this research suggest increasing the target concentration, along with refining the selected oligonucleotide pool [75].

The modeling of SELEX against complex targets has shown that increasing selection rigidity (including reducing the target concentration) does not always favor selection [77], and aptamers are better selected with greater target abundance in the mix [78]. Besides this, simulations of subtractive SELEX have indicated that negative selection rounds provide better results with a higher concentration of counter-targets [79].

Mathematical models can never take into account all possible experimental nuances, and this is a possible reason for the disagreement among the conclusions derived from different computational experiments. A few experimental works have also derived optimal target concentrations. In cell SELEX, ligands were preferably isolated in the presence of more abundant proteins, although even these ligands did not exhibit the best affinity to their targets [8]. A higher concentration of the counter-target has also been recommended for successful negative selection [8]. However, at the same time, too high a density of the immobilized target can result in the isolation of lower-affinity oligonucleotides as a result of cooperative binding effects [80]. The optimization of immobilized target protein concentration is advised for optimal selection because it enables the prevention of possible interactions between one oligonucleotide and two or more target molecules and, at the same time, uses the maximum target concentration for retrieving the maximum quantity of ligands in a selection cycle [7]. No systematic experimental evaluation of the effect of target concentration on selection results was found in the literature. In some works, a 1:100 or 1:1000 aptamer-to-target ratio has been recommended [7]. The variation in target concentration has been used as an instrument for optimizing the selection of an RNA aptamer of the neuropeptide Substance P [42]. During the course of selection, the target concentration was decreased, and three different concentrations were tested in parallel to enable the optimal PCR amplification of the selected RNA pool. Among the concentrations tested, the optimal peptide concentration was the highest in the initial cycles, the lowest in the middle cycles, and then medial in the last selection cycles. Analysis of the provided experimental data allows for the conclusion that a higher concentration always resulted in a larger amount of isolated RNA, but nothing can be concluded about its affinity to the target [42]. Therefore, a detailed experimental evaluation of the role of target concentration in the selection process is needed, because it could produce an instrument for tuning the affinity of aptamers isolated in SELEX experiments.

### 4.2. Incubation Conditions and the Separation of Target-Bound and Unbound Oligonucleotides

The method that is used to select target-binding oligonucleotides from the rest of the pool is a key point of SELEX. Classically, the target molecule is immobilized onto a carrier. In this case, nucleic acid ligands that interact with the target can be physically separated from non-interacting oligonucleotides, which stay in the solution above the carrier. After this, target-bound molecules can be eluted and amplified. The first SELEX experiments were performed using targets immobilized on a nitrocellulose filter [5] and agarose columns [4]. Since 1990, numerous other binding and separation techniques have been invented. Many reviews on SELEX protocol modifications have been published (e.g., [19,81,82,83,84,85,86,87]). Different carriers can be used for target immobilization to achieve easier and more effective separation of ligand–target complexes. The range of SELEX targets has been expanded to cells and small molecules, and these specific targets require special protocol modifications. Cell-SELEX is now almost a separate technology with its own progress [88]. For small molecules, target immobilization is substituted by oligonucleotide library fixation in the Capture-SELEX protocol [89]. Immobilization-free SELEX protocols, such as capillary electrophoresis or sol–gel-based separations, are now available for targets of various sizes [86]. The choice of a particular selection protocol thus depends on the target’s nature and size, on the intended aptamer application, and on the techniques and equipment available in the lab.

To our knowledge, very little research has been devoted to the experimental comparison of different selection strategies for SELEX. Selections of an RNA aptamer against a purified and cell-expressed protein target were compared for tenascin-C [31]. Both protocols enabled the isolation of aptamers with similar sequences, but in the cell-SELEX pool, evolution was retarded because of the high background binding of nucleic acids to the cells instead of the purified proteins. The speed of aptamer isolation was increased by switching the target from cells to the purified protein to ultimately generate a crossover selection scheme. Two additional selection cycles against isolated tenascin-C resulted in a great increase in pool affinity. Cell-SELEX offers the advantage of selecting aptamers of a target in its native matrix. The authors indicated that the success of cell-SELEX appeared to be potentially due to the high abundance of the particular target molecules on the surface of the cells. A similar experiment to compare different selection strategies was performed during the development of an RNA aptamer against membrane receptor tyrosine kinase RET^C634Y^ [8]. Selections against living cells resulted in the isolation of some aptamers with affinity to the target, but most of the pool was enriched with oligonucleotides binding to other, more abundant proteins on the surface of the cell. Aptamers easily isolated during selection against the purified protein were unable to recognize the target expressed in the cell membrane. Crossover selection, switching from cells to the purified enzyme, was also applied, and this strategy allowed for the attainment of aptamers, but they had poorer binding properties compared with cell selections [8]. Mondal et al. used a combination of three separation methods for each selection cycle during aptamer isolation against enterotoxin B from *Staphylococcus aureus* (Ni-NTA affinity column-, nitrocellulose membrane-, and microtiter plate-based separations) and compared this combined protocol with selections using each separation method individually [90]. After six selection cycles, the ratio of target-binging oligonucleotides for a retained ssDNA pool using a combined selection strategy was 51.2%, while that for the individual strategies was less than 10%. So far, the proposed strategy has allowed for faster isolation of aptamers with fewer selection cycles [90]. These studies especially underline the significance of an appropriate selection strategy design for the success of SELEX experiments. Another example of the importance of the partition approach is DNA aptamer selection against xanthine [6]. An immobilization-free sol–gel-based separation method enabled the isolation of aptamers, while SELEX against xanthine covalently linked to polymer resin failed [6].

Most efforts to evaluate the selection progress in selection designs are applied to improve the partition efficiency of the selection. Better partition efficiency results in fewer selection cycles required for the isolation of an aptamer with the desired affinity, because higher background binding decelerates the enrichment of the selected nucleic acid pool in each selection cycle. This has been proved by both mathematical analysis and experiments. In a modeling study, Wang et al. mathematically examined the role of background binding during selection progress [75]. The authors demonstrated that slower enrichment due to high background binding resulted in a higher optimal target concentration [75]. A comparative simulation was carried out for two selections under different conditions: Microfluidic SELEX with high separation efficiency (low background elution) and SELEX on a nitrocellulose membrane with high background elution. Six rounds of selection were examined. The results revealed that nitrocellulose membrane-based SELEX was much more sensitive to improper target concentration. Pool enrichment was much faster for microfluidic SELEX, and the affinity of the resulting pool was 30 times higher for microfluidic SELEX than for the nitrocellulose membrane [75]. Studies with other mathematical models have also highlighted that lower background binding is necessary to minimize the number of selection cycles [71,78]. Ouellet et al. experimentally examined the influence of partition efficiency on the bulk *K_D_* value of the selected DNA pool during the development of a Hi-Fi SELEX protocol [7]. Three cycles of selection were performed with two types of DNA libraries against thrombin immobilized on either magnetic beads or Nunc well plates that were specially treated in order to diminish the background binding of ssDNA. The affinity of the resulting pool in both cases was one order of magnitude lower for the selection scheme with the higher partition efficiency [7].

Another important issue of selection design is the introduction of counter and negative selection steps. Negative selection is the selection against non-target components that are present in the target sample; such components include selection buffer and unmodified carrier (naked nitrocellulose membrane or activated magnetic beads). Counter selection is performed against some substances with structures similar to the target. It is especially relevant for the isolation of aptamers against a target in a complex matrix, such as membrane proteins or receptors on the surface of the cell. It is not surprising that the significance and power of negative selection have been demonstrated using cell-SELEX experiments. Hamula et al. compared selections against *Streptococcus pyogenes* under the same conditions with and without counter selection and reasoned that the introduction of counter selection resulted in the reduction in the SELEX cycle number for obtaining a target-specific aptamer pool [9]. Implementation of counter selection in SELEX sometimes results in the quite fascinating ability of aptamers to discriminate between very similar targets [91].

Thus, the binding and separation step in the SELEX procedure can influence the selection outcome in all possible aspects. The overall selection success becomes possible with an appropriate selection design that considers the target’s nature. The minimization of selection cycles can be achieved with higher separation efficiency, while negative and counter selections contribute to aptamer specificity.

## 5. Amplification

Correct amplification of each enriched DNA pool during SELEX drives the overall success of aptamer selection. Selection usually starts with a highly diversified ssDNA pool in which target-binding sequences are very rare and mostly unique. It is desirable that each oligonucleotide selected after the target-binding–partition step be amplified with high efficiency, and the amplification rate should be the same for all the sequences in the DNA pool. Mathematical analysis has derived that the number of amplification cycles is dependent on target concentration, and a lower target concentration requires more amplification cycles [74]. Notably, as many PCR cycles as possible should be performed. However, in practice, many experimental pitfalls are associated with the PCR amplification step. Random DNA libraries tend to produce byproducts during PCR amplification as a result of high heterogeneity of the template DNA, leading to the occasional primer–product and product–product hybridization [92]. Recombination of homologous regions during PCR also results in byproduct formation [93]. In addition, GC- and AT-rich sequences, along with highly structured oligonucleotides, are not amplified as well as other sequences [93,94], and shorter sequences are amplified better than larger fragments [93]. In light of this PCR bias, sequences are never equally amplified. Each amplification cycle shifts the sequence representativeness of the DNA pool [95]. 

The optimization of PCR conditions can improve amplification performance. First, the optimization of amplification conditions is required. DNA overamplification should be avoided. Typically, the maximum yield of the correct dsDNA product can be achieved with few amplification cycles (6–10), and then the quantity of the target DNA is reduced with each new cycle while byproducts accumulate [92]. Thus, the number of amplification cycles should be optimized. Increasing the primer concentration [96], designing appropriate primers [38], using less bias-prone DNA polymerase [97], and lowering the template concentration can help to achieve a higher amplification rate. Optimal conditions, especially the number of cycles, should be screened for each new enriched DNA pool after each selection step during SELEX.

Library design (both library length and constant regions design) affects PCR amplification efficiency. Musheev and Krylov have experimentally demonstrated that byproduct formation for a 122-nt-long DNA library starts 2–3 cycles earlier than for an 81-nt-long matrix, and amplification of two templates of nearly the same length, but with different primer-binding sites, resulted in a two-fold difference in dsDNA product yield [27].

Byproduct formation in standard PCR is driven by the heterogeneity of nucleic acid libraries. In the reannealing step, DNA chains hybridize partially but not fully, because there is a small chance that complementary strands meet each other in a solution. This problem can be avoided with the implementation of emulsion PCR. It this case, a water-in-oil emulsion is obtained for the PCR master mix. Each small droplet of the emulsion serves as a separate microreactor in which a few or a single nucleotide is isolated and amplified so that no mispairing is possible, and amplification bias is avoided. A few protocols for ePCR for random library amplification have been developed [92,93,98,99,100]. Emulsions can be generated using magnetic stirrers [101], vortexes [98], or microfluidic chips [102]. Emulsion generation with a microfluidic chip is rather convenient because it allows for simple droplet size control [102]. Different oil phases result in different emulsion stabilities; more DNA polymerase and the addition of BSA are required for DNA amplification in emulsions because proteins accumulate at the oil–water interface [103]. The template concentration also needs to be optimized with respect to the droplet size to achieve single-molecule compartmentalization [103]. With too high a number of different copies in a droplet, byproducts are formed during amplification [7,103]. A commercially available droplet digital PCR (ddPCR) system produced by Bio-Rad is often used in modern research to achieve maximum PCR performance [7,11]. With ePCR or ddPCR, the number of amplification cycles can be increased without product loss and with minimized amplification bias [11,100]. Another benefit of ePCR amplification is the formation of homoduplexes of dsDNA products that can be easily transformed to ssDNA using λ exonuclease, while solution PCR generates many heteroduplexes, which cannot be processed with the enzyme [7].

ePCR/ddPCR and solution PCR methods for DNA amplification have been compared in complex SELEX experiments. The diversity of round-by-round selected DNA/RNA pools is strongly preserved with the use of ePCR amplification; this was demonstrated using melting curve analysis and fluorescent quantification of amplified DNA using diversity DNA standards [7] and by high-throughput sequencing [11,100]. Low-abundance sequences did not survive solution PCR amplification, and a significant drop in pool diversities was detected in the early stages of selection [11,100]. Interestingly, high-abundance candidate RNA aptamers of the CCR7 protein isolated after seven SELEX rounds with the assistance of solution and ePCR were different, except for just one overlapping sequence. Even more interesting is that high-abundance sequences derived from solution PCR-assisted SELEX exhibited higher affinity to the target-expressing cells than the most prevalent sequences in ePCR-assisted selection [11]. In another ePCR-assisted SELEX experiment, high-affinity hIL-10RA and h4-1BB protein-binding aptamers were also not the most abundant sequences, but their enrichment rate was higher in comparison with other sequences [100]. This suggests that end-point selected pool sequencing is suitable for aptamer isolation using solution PCR-assisted SELEX, while ePCR-aided selections require round-by-round sequence tracking, which can only be achieved with high-throughput sequencing supported by deep bioinformatics analysis. At the same time, a few attempts to isolate ABH2 protein-binding aptamers using nonequilibrium capillary electrophoresis of equilibrium mixtures were reported to have failed until ePCR was incorporated into the SELEX procedure [104], which is possibly explained by the ability of ePCR to amplify rare but high-affinity sequences.

For RNA SELEX, PCR bias can be omitted with the substitution of PCR amplification by transcription amplification. Tsuji et al. successfully applied this approach, called SELEX-T, to isolate RNA aptamers that bind to macrophage migration inhibitory factor, while no aptamers against this target could be isolated with the classical SELEX protocol [105]. The authors attributed this success to the elimination of PCR bias [105].

In summary, the PCR amplification protocol contributes strongly to SELEX results in terms of the overall selection success and the affinity of isolated sequences. ePCR offers the elimination of PCR bias and the preservation of oligonucleotide library diversity, but its protocol is more expensive and extensive, and a large number of selected sequences are not easy to analyze.

## 6. Pool Conditioning

The amplified DNA pool further undergoes conditioning: Transcription to RNA for RNA SELEX and ssDNA regeneration for DNA SELEX. This step is crucial in the SELEX procedure, since the quantity and quality of RNA or ssDNA obtained can greatly influence the success of the evolution of aptamers.

### 6.1. Transcription to RNA

The amplified dsDNA pool is usually transformed into RNA using RNA polymerase. Some technical points should be addressed in this process. As already mentioned above, shorter DNA fragments are transcribed to RNA with higher efficiency and fewer errors [23,24]. A nucleotide bias against the inclusion of adenine was observed for an RNA library during in vitro evolution, and this bias was shown to be target-independent [106]. This means that the enrichment of an RNA library with some GC-rich sequences is not equal to enrichment with target-binding sequences.

### 6.2. ssDNA Regeneration

Four methods are commonly used for ssDNA regeneration from the dsDNA PCR products: Separation using the streptavidin–biotin interaction coupled with magnetic beads, size separation of DNA strands by denaturing PAGE, asymmetric PCR, and enzymatic digestion of the undesired strand [107]. The evolution process itself is believed to be unaffected by the method of ssDNA regeneration [107], but DNA loss and poor library quality can result in the failure of aptamer selection [108]. So, many researchers have concentrated on comparing these four methods and their optimization to achieve maximum ssDNA recovery.

Magnetic bead-assisted separation of DNA strands employs the tight interaction between streptavidin and biotin. Biotin is usually introduced to the 5’-end of the undesired DNA strand during the PCR amplification step with the aid of a biotinylated reverse primer. The dsDNA product is then captured by streptavidin-covered magnetic beads. Under denaturing conditions, such as high temperatures or an alkaline pH, the DNA strands are dehybridized, and the reverse strand can be removed with the beads by applying a magnet. Although relatively simple, this protocol is rather expensive because of the use of labeled primers and magnetic beads. A significant drawback to this method is the possibility of streptavidin dissociation from the surface of the beads, which can even result in false selection and overall SELEX failure [109]. Denaturation of DNA strands with alkaline pH instead of temperature [110], or with other milder conditions [111], seems to reduce streptavidin leakage. A microfluidic dialysis device for the extraction of ssDNA employing streptavidin-covered magnetic beads was developed, and it enabled the recovery of more pure ssDNA product compared with manual biotin–streptavidin-based DNA separation [112].

Separation of DNA strands using denaturing PAGE demands the use of specially modified primers in the DNA amplification step. Usually, the backward primer is elongated to make the anti-sense strand longer than the sense strand. In [113], two types of modified backward primers were compared: one with a polynucleotide tail added with a hexaethylene spacer and another with a directly added CG-rich polynucleotide tail to form a strong stem–loop structure. The first one was shown to ensure better ssDNA recovery, but PEG-modified primers are costly and can perform poorly in PCR [114]. The backward primer can also be modified with acrydite to immobilize it in the gel, while the sense DNA strand migrates during electrophoresis [114]. The authors stated that this method is much cheaper compared with all of the others [114]. The main disadvantage of PAGE separation is a relatively low ssDNA yield, along with the labor and time intensity of the method.

Enzymatic digestion of the undesired strand can be performed using different exonucleases, of which λ exonuclease and T7 gene 6 exonuclease are the most popular. When employing T7 gene 6 exonuclease, the sense DNA strand is protected by 5’-phosphotlyolate, which is introduced with a chemically-modified forward primer in PCR. The use of λ exonuclease also demands modification of the backward primer used in PCR amplification with 5’-phosphate because it specifically hydrolyzes a phosphorylated DNA strand. λ Exonuclease can also destroy the sense non-phosphorylated DNA strand and target ssDNA by its side activity [115]. The sense DNA strand can be protected from degradation during λ exonuclease treatment by the incorporation of Cy3 or Cy5 fluorescent labels at its 5’ end [116]. After digestion, the removal of the enzyme and its working buffer is required. Enzymes are rather expensive themselves, and this method of ssDNA regeneration is especially demanding for proper DNA library amplification without the formation of any byproducts and partially hybridized duplexes. Nonetheless, enzymatic ssDNA regeneration provides a high recovery rate of ssDNA, especially when using optimized conditions for DNA amplification and hydrolysis [117].

Asymmetric PCR directly generates ssDNA through the use of unequal concentrations of primers. After amplification, the ssDNA product is purified from the reaction mix components. Efficient ssDNA yield can be achieved by the thorough optimization of amplification conditions, such as the primer ratio, annealing temperature, quantity of template DNA, and Mg^2+^ concentration [108,118,119]. Optimization of the conditions for asymmetric PCR is recommended for each SELEX cycle individually [120]. The use of special blocking oligonucleotides is advised to reduce the formation of PCR byproducts [121]. Removal of ineluctable dsDNA product is also desirable because the anti-sense strand can occasionally hybridize and mute possible aptamers [122]; therefore, asymmetric PCR is often combined with other ssDNA regeneration techniques, such as gel purification [118] or enzymatic digestion [108]. Additionally, asymmetric PCR can be combined with the biotin–streptavidin-based method. For example, the capture and release protocol, in which target-free ssDNA is collected by using a capture oligonucleotide that covers the streptavidin-coated magnetic beads and then eluted in specially optimized mild conditions to minimize streptavidin leakage, has been successfully applied for aptamer selection [111]. The dsDNA product of asymmetric PCR can also be removed using streptavidin-coated magnetic beads without an elution step if the backward chain contains a biotin label [123].

Several research works have compared the effectiveness of ssDNA regeneration methods to determine the best one. In one such study, the biotin–streptavidin interaction and PAGE purification-based methods were shown to yield less than 30% ssDNA recovery, while the regeneration of ssDNA using λ exonuclease provided more than a 60% ssDNA yield [122]. λ Exonuclease digestion of dsDNA was shown to be more effective than T7 λ exonuclease hydrolysis of the anti-sense strand, and both enzymatic methods were proved to produce higher ssDNA content than the magnetic bead-based method [124]. Svobodová et al. compared magnetic bead separation, enzymatic digestion with T7 Gene 6 and λ exonucleases, asymmetric PCR, and asymmetric PCR assisted with enzymatic hydrolysis of the dsDNA product. This last protocol provided the best yield of ssDNA (83.9 ± 6.2) [108]. 

It is worth mentioning that successive selection is sometimes possible without withdrawing the anti-sense strand. DNA strands can be thermally denatured. With fast cooling, the strands do not rehybridize, and the denatured pool is evolved in the target-binding process of the SELEX cycle [125]. In this case, some anti-sense strands can even be isolated as aptamers [95,125]. The heat denaturation method for ssDNA regeneration was compared with gel purification and magnetic bead-assisted SELEX experiments. It enabled the isolation of a target-affinity aptamer pool, but selection took 20 rounds of SELEX instead of 8 rounds with the use of gel purification [9].

Different methods for ssDNA pool regeneration mostly compete for maximum product yield, a lower price, and less time input, but, if correctly performed, each of them can be used in SELEX.

## 7. Monitoring of Selection Progress and Selection Cycle Number

The design of SELEX experiments almost always aims to minimize the number of selection cycles, because each selection cycle is rather expensive, time-consuming, and laborious, and each researcher is interested in the number of selection rounds that is sufficient to ensure the success of aptamer isolation. 

Some basic information on the sufficient number of selection cycles has been mathematically derived from SELEX models and simulations. First, the ideal partitioning of target-bound and unbound oligonucleotides and the perfect amplification of selected pools are key for minimizing the number of selection cycles [75]. Mathematical analysis of SELEX has revealed that, theoretically, 2–3 cycles of selection are enough for successive aptamer isolation if the appropriate target concentration is used [71]. One-pot selection against a few targets demands more cycles than selection against a single target [78]. In practice, SELEX experiments are rarely performed with so few rounds, and usually, 5–15 cycles are needed, possibly because of non-ideal partition and pool amplification bias.

To understand how many SELEX cycles should be performed in a particular selection experiment without superfluous rounds, different methods of selection progress monitoring are incorporated into the SELEX protocol. SELEX progress monitoring can also provide information for the on-time control of selection, for example, to decide on whether to introduce negative selection rounds or increase selection pressure.

Methods for SELEX monitoring can roughly be divided into two groups. The first one is the affinity estimation of the oligonucleotide pool selected in each round. For this purpose, the direct measurement of the binding/dissociation constant of the pool can be performed using methods such as surface plasmon resonance (SPR) [126,127], enzyme-linked oligonucleotide assay (ELONA) [90], fluorescent dye-linked aptamer assay (FLAA) [128], and many others, which mostly depend on the specific target and the intended use of the aptamer. In this case, the selection is terminated when the bulk affinity of the pool has stopped increasing or has reached a desired value. As a kind of pool affinity assessment, the quantity of target-bound DNA/RNA after each selection cycle or the percent of the target-bound oligonucleotide pool can be measured using fluorescent labeling of the library [89,129] or qPCR [130]. The selection is accomplished when the number of target-bound oligonucleotides stops increasing. Special similar affinity-assessing techniques were developed for cell-SELEX: Flow cytometry [131], fluorescent imaging [131,132], modifications of enzyme-linked oligonucleotide assays [133,134], qPCR adaptations [135], and fluorescent-activated cell sorting (FACS) [136]. Another approach to selection progress control is the monitoring of pool enrichment. Under selection pressure, the initial highly diverse oligonucleotide pool undergoes evolution, during which it is enriched with target-binding species, while non-binders vanish through the selection process. Thus, the diversity of the selected pools is reduced with each selection cycle, and the end of selection is indicated by the pool diversity ceasing to change. Various instrumental methods have been implemented to track diversity changes. Most popular are those based on melting and remelting curve analysis of the amplified DNA pool [13,14,15,137], and NMR [138] and denaturing HPLC-based [139] techniques have been developed. Besides these processes, high-throughput sequencing (HITS) can be applied for evolution tracking [95,138,140,141].

Some aptamer selection studies have intended to compare the performance of different methods for SELEX progress monitoring. Five different methods for monitoring SELEX progress by using magnetic bead-immobilized streptavidin as a model target were experimentally compared by Mencin et al. [140]. As methods of assessing pool diversity and target-bound ssDNA quantification, with fluorescent labeling and qPCR used as affinity trackers, melting curve analysis and fragment length polymorphism analysis were compared and verified by the direct measurement of the bulk *K_D_* of the DNA pools obtained at different selection stages, and Sanger sequencing was used for some pools. All the methods showed good correlation to each other and were consistent with the selection results identified by sequencing and *K_D_* measurements. qPCR method with SYBR Green I as a fluorescent reporter has a limitation in the quantification of random libraries undergoing evolution, because its results are dependent on the GC content of the quantified DNA [140]. In this research, nine cycles of selection were performed. The diversity of the pool dropped significantly at round 4. The quantity of target-bound ssDNA stopped growing after the sixth cycle, while the pool *K_D_* that was estimated at rounds 4, 7, and 9 decreased for each cycle. Sequencing of the pools eluted in cycles 4, 7, and 9 showed that the main binding motif could be recognized in cycle 4, and its fraction increased in cycle 9 dramatically, while one more binding motif was lost to this last cycle [140].

In another model of aptamer selection against streptavidin [95], the sequence diversity of the pool was analyzed with a calibrated diversity assay for nucleic acid libraries using a diversity standard of random oligonucleotides [15]. In this experiment, the researchers also observed a drop at round 4 that was consistent with the increase in pool affinity, which was measured directly by a fluorescent dye-linked aptamer assay (FLAA). High-throughput Illumina sequencing was applied to track pool DNA evolution during selection, and its results indicated that effective binders could be recognized at round 3 by assessing the enrichment rates for individual clones.

NMR spectra analysis was used as a tool for SELEX progress monitoring and was also consistent with SPR affinity measurement of the selected pools and HTS data [138]. The AML1 Runt domain served as a target for the selection of RNA aptamers. At the fourth round of selection, pool affinity to the target protein started increasing, NMR spectra analysis revealed the loss of pool diversity, and HTS results proved the appearance of one predominant aptamer sequence. During the next four selection rounds, the pool was enriched with two more aptamer sequences, and it was reflected by both an increase in affinity and a loss of diversity [138]. Remarkably, the three predominant aptamer sequences were not the best binders to the target, and the highest affinity was detected for another, less abundant sequence [142].

In the selection of DNA aptamers to tobramycin using the Capture-SELEX protocol, the affinity of the pool was detected at cycle 8, while HTS results indicated library enrichment at cycles 4/6, and target-binding sequences could be recognized at round 2 using sequencing data [126].

In all of the described experiments, the employment of affinity measurement or DNA quantification for SELEX monitoring resulted in a slightly higher number of selection iterations compared with the use of enrichment assessment methods, and in all cases, HTS data enabled very early candidate identification. The method used for SELEX monitoring can thus influence the number of cycles needed for aptamer isolation but does not predictably alter the affinity of the resulting aptamers. Better monitoring is enabled with a combination of different methods [140]. The choice of a particular method for selection control depends mainly on the aims of the experiment, equipment availability, and cost-effectiveness.

## 8. Sequencing and Candidate Aptamer Identification (Sanger vs. Next-Generation Sequencing (NGS))

The aptamer isolation process retrieves a nucleic acid library enriched with sequences binding specifically with a target. In the classic method, the resulting enriched nucleic acid pool is cloned, and 30–100 clones are than Sanger-sequenced with the aim of determining a few aptamer candidates for further detailed characterization. Cluster analysis assists in the identification of aptamer candidates. Usually, the most abundant species or representatives of the largest clusters among the sequenced pool are believed to be potential aptamers. Many aptamers have been isolated and characterized using this understandable approach.

With the great progress of next-generation sequencing (NGS) technologies in the last decade, Sanger sequencing in the field of aptamer development is being driven out by high-throughput sequencing. A few research projects have addressed in detail the difference between these two sequencing approaches. 

Schütze et al. performed 10 rounds of aptamer selection from a DNA library using streptavidin-covered magnetic beads as a model target [95]. After the final SELEX round, 25 clones were Sanger-sequenced, and pools from each round were sequenced using the Illumina platform. Sanger sequencing identified some aptamers. HTS revealed that the best binders identified by Sanger sequences started accumulating during very early rounds and could be already identified in the third round of selection. The most abundant sequence identified by NGS for round 10 did not show the best binding to the target [95]. Authors suggested that prevalent sequences originated from PCR bias but not target-induced evolution [95].

Comparison of end-point Sanger sequencing and round-by-round HTS was performed by Civit et al. during the selection of DNA aptamers that could recognize breast cancer cells [143]. Enrichment rate scoring allowed for the identification of the best binders. These aptamers were not identified with Sanger sequencing, possibly because of a relatively low abundance in the resulting pool. Besides this, the HTS results indicated that library enrichment started during the fifth SELEX cycle, and the aptamers could be recognized in much earlier rounds (7 instead of 12) [143].

Stoltenburg et al. used medium-throughput sequencing to refine the results of a classical DNA SELEX against Protein A [144]. One aptamer sequence was derived from an experiment using Sanger sequencing [145]. The same resulting pool was later sequenced with a Roche 454 platform [144]. Compared with Sanger sequencing, NGS identified two more families of additional target-specific sequences, but the aptamer with the best binding properties was identified by both Sanger and next-generation sequencing [144]. In contrast to most of the other NGS-employing research, this work used NGS only for end-point sequencing of a resulting nucleic acid pool instead of complex cycle-by-cycle analysis of each pool, which is a compromise between the lack of information in Sanger sequencing and the analysis complexity in round-by-round HTS.

Taken together, these results suggest that NGS offers benefits over Sanger sequencing with respect to the early identification of candidate aptamers. Besides this, high-throughput sequencing is an outstanding technique for SELEX control because it provides maximum information on pool evolution and is very helpful for understanding the SELEX process and progress. HTS can detect binders within 2–3 selection cycles on the basis of enrichment rate scoring for individual sequences [95,126,138,140,141]. The other side of this tool is the huge amount of data produced by HTS. This requires special analysis methods based on new algorithms and specialized software [146], demanding more intensive bioinformatics research.

## 9. Aptamer Characterization

Appropriate characterization of the selected aptamer candidates is an important issue of SELEX experiments. Though carefully isolated and reasonably selected after sequencing analysis, the candidates do not always prove target binding. This can happen due to several reasons: The change of experimental conditions for binding assay or intended application of the aptamer in comparison to the selection strategy design; the incorrect representation of library members induced by PRC bias, resulting in improper identification of the candidates; the contribution of the fixed regions to the formation of aptamer structure in the case of truncated sequences; the possibility that two sequences work synergetically. McKeague et al. performed a comparison of different binding assays for ochratoxin A aptamer with its target [147]. Results of eight methods for binding evaluation were inconsistent with each other. A combination of a few assays is advised for correct measurement of aptamer target-binding [147].

Besides determination of target-binding characteristics like *K_D_* or *IC_50_*, an aptamer should be characterized with target specificity and sustainability to change of environment like buffer pH and composition, salt concentration, temperature, and any other conditions especially relevant for intended aptamer application. And, finally, for any assay negative and non-binding controls implementation is obligatory for correct aptamer characterization.

## 10. Conclusions

The design of a SELEX experiment contributes strongly to the success of aptamer development. A large and maximally structurally diverse starting library, the appropriate design of the binding and separation procedure, optimized amplification, and pool regeneration protocols contribute to the ability to isolate aptamers. The chance of isolating aptamers with higher affinity increases with higher structural complexity of the initial nucleic acid library and with the appropriate target concentration. Chemically modified oligonucleotide libraries appear a source of high affinity aptamers with increased stability. The specificity of the selected aptamers can be tuned using negative and counter selections. The number of selection cycles, and thus time and effort required for aptamer isolation, can be minimized using separation methods with better partition efficiency, enrichment-assessing methods for selection progress monitoring, and especially by introducing NGS to selection experiments. NGS expansion to SELEX procedures offers the prospect of a deeper understanding of the aptamer selection process. Detailed characterization of the selected aptamers is an unalienable part of any SELEX experiment that finally terms the success of selection experiment.

## References

[1] Kaur H., Bruno J.G., Kumar A., Sharma T.K. (2018). Aptamers in the Therapeutics and Diagnostics Pipelines. Theranostics.

[2] Ilgu M., Nilsen-Hamilton M. (2016). Aptamers in analytics. Analyst.

[3] McKeague M., Derosa M.C. (2012). Challenges and opportunities for small molecule aptamer development. J. Nucleic Acids.

[4] Ellington A.D., Szostak J.W. (1990). In vitro selection of RNA molecules that bind specific ligands. Nature.

[5] Tuerk C., Gold L. (1990). Systematic evolution of ligands by exponential enrichment: RNA ligands to bacteriophage T4 DNA polymerase. Science.

[6] Bae H., Ren S., Kang J., Kim M., Jiang Y., Jin M.M., Min I.M., Kim S. (2013). Sol-Gel SELEX Circumventing Chemical Conjugation of Low Molecular Weight Metabolites Discovers Aptamers Selective to Xanthine. Nucleic Acid Ther..

[7] Ouellet E., Foley J.H., Conway E.M., Haynes C. (2015). Hi-Fi SELEX: A High-Fidelity Digital-PCR Based Therapeutic Aptamer Discovery Platform. Biotechnol. Bioeng..

[8] Pestourie C., Cerchia L., Gombert K., Aissouni Y., Boulay J., De Franciscis V., Libri D., Tavitian B., Ducongé F. (2006). Comparison of Different Strategies to Select Aptamers Against a Transmembrane Protein Target. Oligonucleotides.

[9] Hamula C.L.A., Peng H., Wang Z., Newbigging A.M., Tyrrell G.J., Li X.F., Le X.C. (2015). The Effects of SELEX Conditions on the Resultant Aptamer Pools in the Selection of Aptamers Binding to Bacterial Cells. J. Mol. Evol..

[10] Hall B., Micheletti J.M., Satya P., Ogle K., Pollard J., Ellington A.D. (2009). Design, Synthesis, and Amplification of DNA Pools for In Vitro Selection. Current Protocols in Nucleic Acid Chemistry.

[11] Takahashi M., Wu X., Ho M., Chomchan P., Rossi J.J., Burnett J.C., Zhou J. (2016). High throughput sequencing analysis of RNA libraries reveals the influences of initial library and PCR methods on SELEX efficiency. Sci. Rep..

[12] Wilson R., Bourne C., Chaudhuri R.R., Gregory R., Kenny J., Cossins A. (2014). Single-Step Selection of Bivalent Aptamers Validated by Comparison with SELEX Using High-Throughput Sequencing. PLoS ONE.

[13] Vanbrabant J., Leirs K., Vanschoenbeek K., Lammertyn J., Michiels L. (2014). reMelting curve analysis as a tool for enrichment monitoring in the SELEX process. Analyst.

[14] Charlton J., Smith D. (1999). Estimation of SELEX pool size by measurement of DNA renaturation rates. RNA.

[15] Schütze T., Arndt P.F., Menger M., Wochner A., Vingron M., Erdmann V.A., Lehrach H., Kaps C., Glökler J. (2010). A calibrated diversity assay for nucleic acid libraries using DiStRO—a Diversity Standard of Random Oligonucleotides. Nucleic Acids Res..

[16] Zuker M. (2003). Mfold web server for nucleic acid folding and hybridization prediction. Nucleic Acids Res..

[17] Vorobyeva M., Davydova A., Vorobjev P., Pyshnyi D., Venyaminova A. (2018). Key Aspects of Nucleic Acid Library Design for in Vitro Selection. Int. J. Mol. Sci..

[18] Sampson T. (2003). Aptamers and SELEX: The technology. World Pat. Inf..

[19] Stoltenburg R., Reinemann C., Strehlitz B. (2007). SELEX—A (r)evolutionary method to generate high-affinity nucleic acid ligands. Biomol. Eng..

[20] Salehi-Ashtiani K., Szostak J.W. (2001). In vitro evolution suggests multiple origins for the hammerhead ribozyme. Nature.

[21] Lozupone C. (2003). Selection of the simplest RNA that binds isoleucine. RNA.

[22] Legiewicz M. (2005). Size, constant sequences, and optimal selection. RNA.

[23] Coleman T.M., Huang F. (2002). RNA-Catalyzed Thioester Synthesis. Chem. Biol..

[24] Coleman T.M., Huang F. (2005). Optimal Random Libraries for the Isolation of Catalytic RNA. RNA Biol..

[25] Conaty J., Hendry P., Lockett T. (1999). Selected classes of minimised hammerhead ribozyme have very high cleavage rates at low Mg2+ concentration. Nucleic Acids Res..

[26] Cowperthwaite M.C., Ellington A.D. (2008). Bioinformatic Analysis of the Contribution of Primer Sequences to Aptamer Structures. J. Mol. Evol..

[27] Musheev M.U., Krylov S.N. (2006). Selection of aptamers by systematic evolution of ligands by exponential enrichment: Addressing the polymerase chain reaction issue. Anal. Chim. Acta.

[28] Bock L.C., Griffin L.C., Latham J.A., Vermaas E.H., Toole J.J. (1992). Selection of single-stranded DNA molecules that bind and inhibit human thrombin. Nature.

[29] Hesselberth J.R., Miller D., Robertus J., Ellington A.D. (2000). In Vitro Selection of RNA Molecules That Inhibit the Activity of Ricin A-chain. J. Biol. Chem..

[30] Berezhnoy A., Stewart C.A., Mcnamara J.O., Thiel W., Giangrande P., Trinchieri G., Gilboa E. (2012). Isolation and Optimization of Murine IL-10 Receptor Blocking Oligonucleotide Aptamers Using High-throughput Sequencing. Mol. Ther..

[31] Hicke B.J., Marion C., Chang Y.F., Gould T., Lynott C.K., Parma D., Schmidt P.G., Warren S. (2001). Tenascin-C Aptamers Are Generated Using Tumor Cells and Purified Protein. J. Biol. Chem..

[32] Pan W., Xin P., Clawson G.A. (2008). Minimal primer and primer-free SELEX protocols for selection of aptamers from random DNA libraries. Biotechniques.

[33] Wen J.D. (2004). Selection of genomic sequences that bind tightly to Ff gene 5 protein: Primer-free genomic SELEX. Nucleic Acids Res..

[34] Legiewicz M., Yarus M. (2005). A More Complex Isoleucine Aptamer with a Cognate Triplet. J. Biol. Chem..

[35] Connell G.J., Illangesekare M., Yarus M. (1993). Three small ribooligonucleotides with specific arginine sites. Biochemistry.

[36] Ellington A.D., Khrapov M., Shaw C.A. (2000). The scene of a frozen accident. RNA.

[37] Pan W., Clawson G. (2009). The Shorter the Better: Reducing Fixed Primer Regions of Oligonucleotide Libraries for Aptamer Selection. Molecules.

[38] Tolle F., Wilke J., Wengel J., Mayer G. (2014). By-Product Formation in Repetitive PCR Amplification of DNA Libraries during SELEX. PLoS ONE.

[39] Yang D.K., Chou C.F., Chen L.C. (2018). Selection of aptamers for AMACR detection from DNA libraries with different primers. RSC Adv..

[40] Boiziau C., Toulmé J.J. (2001). A Method to Select Chemically Modified Aptamers Directly. Antisense Nucleic Acid Drug Dev..

[41] Vater A. (2003). Short bioactive Spiegelmers to migraine-associated calcitonin gene-related peptide rapidly identified by a novel approach: Tailored-SELEX. Nucleic Acids Res..

[42] Eulberg D. (2005). Development of an automated in vitro selection protocol to obtain RNA-based aptamers: Identification of a biostable substance P antagonist. Nucleic Acids Res..

[43] Jarosch F. (2006). In vitro selection using a dual RNA library that allows primerless selection. Nucleic Acids Res..

[44] Lai Y.T., DeStefano J.J. (2011). A primer-free method that selects high-affinity single-stranded DNA aptamers using thermostable RNA ligase. Anal. Biochem..

[45] Tsao S.M., Lai J.C., Horng H.E., Liu T.C., Hong C.Y. (2017). Generation of Aptamers from A Primer-Free Randomized ssDNA Library Using Magnetic-Assisted Rapid Aptamer Selection. Sci. Rep..

[46] Shtatland T. (2000). Interactions of Escherichia coli RNA with bacteriophage MS2 coat protein: Genomic SELEX. Nucleic Acids Res..

[47] Hamm J. (1996). Characterisation of Antibody-Binding RNAs Selected from Structurally Constrained Libraries. Nucleic Acids Res..

[48] Ouellet E., Lagally E.T., Cheung K.C., Haynes C.A. (2014). A simple method for eliminating fixed-region interference of aptamer binding during SELEX. Biotechnol. Bioeng..

[49] Oh S.S., Plakos K., Lou X., Xiao Y., Soh H.T. (2010). In vitro selection of structure-switching, self-reporting aptamers. Proc. Natl. Acad. Sci. USA.

[50] Gevertz J. (2005). In vitro RNA random pools are not structurally diverse: A computational analysis. RNA.

[51] Davis J.H., Szostak J.W. (2002). Isolation of high-affinity GTP aptamers from partially structured RNA libraries. Proc. Natl. Acad. Sci. USA.

[52] Zhu L., Li C., Zhu Z., Liu D., Zou Y., Wang C., Fu H., Yang C.J. (2012). In Vitro Selection of Highly Efficient G-Quadruplex-Based DNAzymes. Anal. Chem..

[53] Luo X., McKeague M., Pitre S., Dumontier M., Green J., Golshani A., Derosa M.C., Dehne F. (2010). Computational approaches toward the design of pools for the in vitro selection of complex aptamers. RNA.

[54] Kim N., Shin J.S., Elmetwaly S., Gan H.H., Schlick T. (2007). RAGPOOLS: RNA-As-Graph-Pools a web server for assisting the design of structured RNA pools for in vitro selection. Bioinformatics.

[55] Carothers J.M., Oestreich S.C., Davis J.H., Szostak J.W. (2004). Informational Complexity and Functional Activity of RNA Structures. J. Am. Chem. Soc..

[56] Kim N., Gan H.H., Schlick T. (2007). A computational proposal for designing structured RNA pools for in vitro selection of RNAs. RNA.

[57] Ruff K.M., Snyder T.M., Liu D.R. (2010). Enhanced Functional Potential of Nucleic Acid Aptamer Libraries Patterned to Increase Secondary Structure. J. Am. Chem. Soc..

[58] Carothers J.M. (2006). Solution structure of an informationally complex high-affinity RNA aptamer to GTP. RNA.

[59] Yang K.A., Pei R., Stefanovic D., Stojanovic M.N. (2012). Optimizing Cross-reactivity with Evolutionary Search for Sensors. J. Am. Chem. Soc..

[60] Trevino S.G., Levy M. (2014). High-Throughput Bead-Based Identification of Structure-Switching Aptamer Beacons. ChemBioChem.

[61] Lipi F., Chen S., Chakravarthy M., Rakesh S., Veedu R.N. (2016). In vitro evolution of chemically-modified nucleic acid aptamers: Pros and cons, and comprehensive selection strategies. RNA Biol..

[62] Ni S., Yao H., Wang L., Lu J., Jiang F., Lu A., Zhang G. (2017). Chemical Modifications of Nucleic Acid Aptamers for Therapeutic Purposes. Int. J. Mol. Sci..

[63] Lapa S.A., Chudinov A.V., Timofeev E.N. (2016). The Toolbox for Modified Aptamers. Mol. Biotechnol..

[64] Diafa S., Hollenstein M. (2015). Generation of Aptamers with an Expanded Chemical Repertoire. Molecules.

[65] Stovall G.M., Bedenbaugh R.S., Singh S., Meyer A.J., Hatala P.J., Ellington A.D., Hall B. (2014). In Vitro Selection Using Modified or Unnatural Nucleotides. Current Protocols in Nucleic Acid Chemistry.

[66] Li M., Lin N., Huang Z., Du L., Altier C., Fang H., Wang B. (2008). Selecting Aptamers for a Glycoprotein through the Incorporation of the Boronic Acid Moiety. J. Am. Chem. Soc..

[67] Imaizumi Y., Kasahara Y., Fujita H., Kitadume S., Ozaki H., Endoh T., Kuwahara M., Sugimoto N. (2013). Efficacy of Base-Modification on Target Binding of Small Molecule DNA Aptamers. J. Am. Chem. Soc..

[68] Gold L., Ayers D., Bertino J., Bock C., Bock A., Brody E.N., Carter J. (2010). Aptamer-Based Multiplexed Proteomic Technology for Biomarker Discovery. PLoS ONE.

[69] Davies D.R., Gelinas A.D., Zhang C., Rohloff J.C., Carter J.D., O’Connell D., Waugh S.M., Wolk S.K., Mayfield W.S., Burgin A.B. (2012). Unique motifs and hydrophobic interactions shape the binding of modified DNA ligands to protein targets. Proc. Natl. Acad. Sci. USA.

[70] Vaught J.D., Bock C., Carter J., Fitzwater T., Otis M., Schneider D., Rolando J., Waugh S., Wilcox S.K., Eaton B.E. (2010). Expanding the chemistry of DNA for in vitro selection. J. Am. Chem. Soc..

[71] Irvine D., Tuerk C., Gold L. (1991). Selexion: Systematic evolution of ligands by exponential enrichment with integrated optimization by non-linear analysis. J. Mol. Biol..

[72] Levine H.A., Nilsen-Hamilton M. (2007). A mathematical analysis of SELEX. Comput. Biol. Chem..

[73] Levine H.A., Seo Y.J. (2015). Discrete Dynamical Systems in Multiple Target and Alternate SELEX. SIAM J. Appl. Dyn. Syst..

[74] Sun F., Galas D., Waterman M.S. (1996). A Mathematical Analysis ofin VitroMolecular Selection – Amplification. J. Mol. Biol..

[75] Wang J., Rudzinski J.F., Gong Q., Soh H.T., Atzberger P.J. (2012). Influence of Target Concentration and Background Binding on In Vitro Selection of Affinity Reagents. PLoS ONE.

[76] Aita T., Nishigaki K., Husimi Y. (2012). Theoretical consideration of selective enrichment in in vitro selection: Optimal concentration of target molecules. Math. Biosci..

[77] Chen C.K. (2007). Complex SELEX against target mixture: Stochastic computer model, simulation, and analysis. Comput. Methods Programs Biomed..

[78] Vant-Hull B., Payano-Baez A., Davis R.H., Gold L. (1998). The mathematics of SELEX against complex targets. J. Mol. Biol..

[79] Chen C.K., Kuo T.L., Chan P.C., Lin L.Y. (2007). Subtractive SELEX against two heterogeneous target samples: Numerical simulations and analysis. Comput. Biol. Med..

[80] Ozer A., White B.S., Lis J.T., Shalloway D. (2013). Density-dependent cooperative non-specific binding in solid-phase SELEX affinity selection. Nucleic Acids Res..

[81] Gopinath S.C.B. (2006). Methods developed for SELEX. Anal. Bioanal. Chem..

[82] Zhuo Z., Yu Y., Wang M., Li J., Zhang Z., Liu J., Wu X., Lu A., Zhang G., Zhang B. (2017). Recent Advances in SELEX Technology and Aptamer Applications in Biomedicine. Int. J. Mol. Sci..

[83] Darmostuk M., Rimpelova S., Gbelcova H., Ruml T. (2015). Current approaches in SELEX: An update to aptamer selection technology. Biotechnol. Adv..

[84] Dong Y., Wang Z., Wang S., Wu Y., Ma Y., Liu J. (2018). Introduction of SELEX and Important SELEX Variants. Aptamers for Analytical Applications.

[85] Zhang Y., Lai B., Juhas M. (2019). Recent Advances in Aptamer Discovery and Applications. Molecules.

[86] Bayat P., Nosrati R., Alibolandi M., Rafatpanah H., Abnous K., Khedri M., Ramezani M. (2018). SELEX methods on the road to protein targeting with nucleic acid aptamers. Biochimie.

[87] Ozer A., Pagano J.M., Lis J.T. (2014). New Technologies Provide Quantum Changes in the Scale, Speed, and Success of SELEX Methods and Aptamer Characterization. Mol. Ther. Nucleic Acids.

[88] Kaur H. (2018). Recent developments in cell-SELEX technology for aptamer selection. Biochim. Biophys. Acta Gen. Subj..

[89] Stoltenburg R., Nikolaus N., Strehlitz B. (2012). Capture-SELEX: Selection of DNA Aptamers for Aminoglycoside Antibiotics. J. Anal. Methods Chem..

[90] Mondal B., Ramlal S., Lavu P.S.R., Murali H.S., Batra H.V. (2015). A combinatorial systematic evolution of ligands by exponential enrichment method for selection of aptamer against protein targets. Appl. Microbiol. Biotechnol..

[91] Srinivasan J., Cload S.T., Hamaguchi N., Kurz J., Keene S., Kurz M., Boomer R.M., Blanchard J., Epstein D., Wilson C. (2004). ADP-Specific Sensors Enable Universal Assay of Protein Kinase Activity. Chem. Biol..

[92] Shao K., Ding W., Wang F., Li H., Ma D., Wang H. (2011). Emulsion PCR: A High Efficient Way of PCR Amplification of Random DNA Libraries in Aptamer Selection. PLoS ONE.

[93] Williams R., Peisajovich S.G., Miller O.J., Magdassi S., Tawfik D.S., Griffiths A.D. (2006). Amplification of complex gene libraries by emulsion PCR. Nat. Methods.

[94] Day D. (1996). Identification of non-amplifying CYP21 genes when using PCR-based diagnosis of 21-hydroxylase deficiency in congenital adrenal hyperplasia (CAH) affected pedigrees. Hum. Mol. Genet..

[95] Schütze T., Wilhelm B., Greiner N., Braun H., Peter F., Mörl M., Erdmann V.A., Lehrach H., Konthur Z., Menger M. (2011). Probing the SELEX Process with Next-Generation Sequencing. PLoS ONE.

[96] Czerny T. (1996). High Primer Concentration Improves PCR Amplification from Random Pools. Nucleic Acids Res..

[97] Van Dijk E.L., Jaszczyszyn Y., Thermes C. (2014). Library preparation methods for next-generation sequencing: Tone down the bias. Exp. Cell Res..

[98] Schütze T., Rubelt F., Repkow J., Greiner N., Erdmann V.A., Lehrach H., Konthur Z., Glökler J. (2011). A streamlined protocol for emulsion polymerase chain reaction and subsequent purification. Anal. Biochem..

[99] Diehl F., Li M., He Y., Kinzler K.W., Vogelstein B., Dressman D. (2006). BEAMing: Single-molecule PCR on microparticles in water-in-oil emulsions. Nat. Methods.

[100] Levay A., Brenneman R., Hoinka J., Sant D., Cardone M., Trinchieri G., Przytycka T.M., Berezhnoy A. (2015). Identifying high-affinity aptamer ligands with defined cross-reactivity using high-throughput guided systematic evolution of ligands by exponential enrichment. Nucleic Acids Res..

[101] Meyerhans A., Vartanian J.P., Wain-Hobson S. (1990). DNA recombination during PCR. Nucleic Acids Res..

[102] Zhu Z., Jenkins G., Zhang W., Zhang M., Guan Z., Yang C.J. (2012). Single-molecule emulsion PCR in microfluidic droplets. Anal. Bioanal. Chem..

[103] Witt M., Phung N.L., Stalke A., Walter J.G., Stahl F., von Neuhoff N., Scheper T. (2017). Comparing two conventional methods of emulsion PCR and optimizing of Tegosoft-based emulsion PCR. Eng. Life Sci..

[104] Yufa R., Krylova S.M., Bruce C., Bagg E.A., Schofield C.J., Krylov S.N. (2015). Emulsion PCR Significantly Improves Nonequilibrium Capillary Electrophoresis of Equilibrium Mixtures-Based Aptamer Selection: Allowing for Efficient and Rapid Selection of Aptamer to Unmodified ABH2 Protein. Anal. Chem..

[105] Tsuji S., Hirabayashi N., Kato S., Akitomi J., Egashira H., Tanaka T., Waga I., Ohtsu T. (2009). Effective isolation of RNA aptamer through suppression of PCR bias. Biochem. Biophys. Res. Commun..

[106] Thiel W.H., Bair T., Wyatt Thiel K., Dassie J.P., Rockey W.M., Howell C.A., Liu X.Y., Dupuy A.J., Huang L., Owczarzy R. (2011). Nucleotide Bias Observed with a Short SELEX RNA Aptamer Library. Nucleic Acid Ther..

[107] Marimuthu C., Tang T.H., Tominaga J., Tan S.C., Gopinath S.C.B. (2012). Single-stranded DNA (ssDNA) production in DNA aptamer generation. Analyst.

[108] Svobodová M., Pinto A., Nadal P., O’ Sullivan C.K. (2012). Comparison of different methods for generation of single-stranded DNA for SELEX processes. Anal. Bioanal. Chem..

[109] Paul A., Avci-Adali M., Ziemer G., Wendel H.P. (2009). Streptavidin-Coated Magnetic Beads for DNA Strand Separation Implicate a Multitude of Problems During Cell-SELEX. Oligonucleotides.

[110] Wilson R. (2011). Preparation of Single-Stranded DNA from PCR Products with Streptavidin Magnetic Beads. Nucleic Acid Ther..

[111] Hamedani N.S., Blümke F., Tolle F., Rohrbach F., Rühl H., Oldenburg J., Mayer G., Pötzsch B., Müller J. (2015). Capture and Release (CaR): A simplified procedure for one-tube isolation and concentration of single-stranded DNA during SELEX. Chem. Commun..

[112] Sheng Y., Bowser M.T. (2014). Isolating single stranded DNA using a microfluidic dialysis device. Analyst.

[113] Liang C., Li D., Zhang G., Li H., Shao N., Liang Z., Zhang L., Lu A., Zhang G. (2015). Comparison of the methods for generating single-stranded DNA in SELEX. Analyst.

[114] Damase T.R., Ellington A.D., Allen P.B. (2017). Purification of single-stranded DNA by co-polymerization with acrylamide and electrophoresis. Biotechniques.

[115] Subramanian K. (2003). The enzymatic basis of processivity in lambda exonuclease. Nucleic Acids Res..

[116] Komarova N.V., Glukhov S.I., Andrianova M.S., Kuznetsov A.E. (2018). Use of the Cy3 and Cy5 Fluorescent Labels to Protect a DNA Strand from Degradation under λ Exonuclease Treatment. Moscow Univ. Chem. Bull..

[117] Citartan M., Tang T.H., Tan S.C., Gopinath S.C.B. (2010). Conditions optimized for the preparation of single-stranded DNA (ssDNA) employing lambda exonuclease digestion in generating DNA aptamer. World J. Microbiol. Biotechnol..

[118] Citartan M., Tang T.H., Tan S.C., Hoe C.H., Saini R., Tominaga J., Gopinath S.C.B. (2012). Asymmetric PCR for good quality ssDNA generation towards DNA aptamer production. Songklanakarin J. Sci. Technol..

[119] He C.Z., Zhang K.H., Wang T., Wan Q.S., Hu P.P., Hu M.D., Huang D.Q., Lv N.H. (2013). Single-primer-limited amplification: A method to generate random single-stranded DNA sub-library for aptamer selection. Anal. Biochem..

[120] Heiat M., Ranjbar R., Latifi A.M., Rasaee M.J., Farnoosh G. (2017). Essential strategies to optimize asymmetric PCR conditions as a reliable method to generate large amount of ssDNA aptamers. Biotechnol. Appl. Biochem..

[121] Tolnai Z., Harkai Á., Szeitner Z., Scholz É.N., Percze K., Gyurkovics A., Mészáros T. (2019). A simple modification increases specificity and efficiency of asymmetric PCR. Anal. Chim. Acta.

[122] Avci-Adali M., Paul A., Wilhelm N., Ziemer G., Wendel H.P. (2009). Upgrading SELEX technology by using lambda exonuclease digestion for single-stranded DNA generation. Molecules.

[123] Zhang Y., Xu H., Zhou H., Wu F., Su Y., Liang Y., Zhou D. (2015). Indirect purification method provides high yield and quality ssDNA sublibrary for potential aptamer selection. Anal. Biochem..

[124] Civit L., Fragoso A., O’Sullivan C.K. (2012). Evaluation of techniques for generation of single-stranded DNA for quantitative detection. Anal. Biochem..

[125] Hung L.Y., Wang C.H., Hsu K.F., Chou C.Y., Lee G.B. (2014). An on-chip Cell-SELEX process for automatic selection of high-affinity aptamers specific to different histologically classified ovarian cancer cells. Lab Chip.

[126] Spiga F.M., Maietta P., Guiducci C. (2015). More DNA-Aptamers for Small Drugs: A Capture-SELEX Coupled with Surface Plasmon Resonance and High-Throughput Sequencing. ACS Comb. Sci..

[127] Jia W., Li H., Wilkop T., Liu X., Yu X., Cheng Q., Xu D., Chen H.Y. (2018). Silver decahedral nanoparticles empowered SPR imaging-SELEX for high throughput screening of aptamers with real-time assessment. Biosens. Bioelectron..

[128] Wochner A., Glökler J. (2007). Nonradioactive fluorescence microtiter plate assay monitoring aptamer selections. Biotechniques.

[129] Stoltenburg R., Reinemann C., Strehlitz B. (2005). FluMag-SELEX as an advantageous method for DNA aptamer selection. Anal. Bioanal. Chem..

[130] Yang D.K., Chen L.C., Lee M.Y., Hsu C.H., Chen C.S. (2014). Selection of aptamers for fluorescent detection of alpha-methylacyl-CoA racemase by single-bead SELEX. Biosens. Bioelectron..

[131] Shangguan D., Bing T., Zhang N. (2015). Cell-SELEX: Aptamer Selection Against Whole Cells. Aptamers Selected by Cell-SELEX for Theranostics.

[132] Kunii T., Ogura S., Mie M., Kobatake E. (2011). Selection of DNA aptamers recognizing small cell lung cancer using living cell-SELEX. Analyst.

[133] Nabavinia M.S., Charbgoo F., Alibolandi M., Mosaffa F., Gholoobi A., Ramezani M., Abnous K. (2018). Comparison of Flow Cytometry and ELASA for Screening of Proper Candidate Aptamer in Cell-SELEX Pool. Appl. Biochem. Biotechnol..

[134] Dastjerdi K., Tabar G.H., Dehghani H., Haghparast A. (2011). Generation of an enriched pool of DNA aptamers for an HER2-overexpressing cell line selected by Cell SELEX. Biotechnol. Appl. Biochem..

[135] Avci-Adali M., Wilhelm N., Perle N., Stoll H., Schlensak C., Wendel H.P. (2013). Absolute Quantification of Cell-Bound DNA Aptamers During SELEX. Nucleic Acid Ther..

[136] Mayer G., Ahmed M.-S.L., Dolf A., Endl E., Knolle P.A., Famulok M. (2010). Fluorescence-activated cell sorting for aptamer SELEX with cell mixtures. Nat. Protoc..

[137] Luo Z., He L., Wang J., Fang X., Zhang L. (2017). Developing a combined strategy for monitoring the progress of aptamer selection. Analyst.

[138] Amano R., Aoki K., Miyakawa S., Nakamura Y., Kozu T., Kawai G., Sakamoto T. (2017). NMR monitoring of the SELEX process to confirm enrichment of structured RNA. Sci. Rep..

[139] Müller J., El-Maarri O., Oldenburg J., Pötzsch B., Mayer G. (2008). Monitoring the progression of the in vitro selection of nucleic acid aptamers by denaturing high-performance liquid chromatography. Anal. Bioanal. Chem..

[140] Mencin N., Šmuc T., Vraničar M., Mavri J., Hren M., Galeša K., Krkoč P., Ulrich H., Šolar B. (2014). Optimization of SELEX: Comparison of different methods for monitoring the progress of in vitro selection of aptamers. J. Pharm. Biomed. Anal..

[141] Gu G., Wang T., Yang Y., Xu X., Wang J. (2013). An Improved SELEX-Seq Strategy for Characterizing DNA-Binding Specificity of Transcription Factor: NF-κB as an Example. PLoS ONE.

[142] Amano R., Takada K., Tanaka Y., Nakamura Y., Kawai G., Kozu T., Sakamoto T. (2016). Kinetic and Thermodynamic Analyses of Interaction between a High-Affinity RNA Aptamer and Its Target Protein. Biochemistry.

[143] Civit L., Taghdisi S.M., Jonczyk A., Haßel S.K., Gröber C., Blank M., Stunden H.J., Beyer M., Schultze J., Latz E. (2018). Systematic evaluation of cell-SELEX enriched aptamers binding to breast cancer cells. Biochimie.

[144] Stoltenburg R., Strehlitz B. (2018). Refining the Results of a Classical SELEX Experiment by Expanding the Sequence Data Set of an Aptamer Pool Selected for Protein A. Int. J. Mol. Sci..

[145] Stoltenburg R., Schubert T., Strehlitz B. (2015). In vitro Selection and Interaction Studies of a DNA Aptamer Targeting Protein A. PLoS ONE.

[146] Blank M. (2016). Next-Generation Analysis of Deep Sequencing Data: Bringing Light into the Black Box of SELEX Experiments. Nucleic Acid Aptamers. Selection, Characterization, and Application.

[147] McKeague M., De Girolamo A., Valenzano S., Pascale M., Ruscito A., Velu R., Frost N.R., Hill K., Smith M., McConnell E.M. (2015). Comprehensive Analytical Comparison of Strategies Used for Small Molecule Aptamer Evaluation. Anal. Chem..

